# Rooted in culture, constrained by means: exploring crops and associated motivations among Masikoro and Antandroy farmers in Southwestern Madagascar

**DOI:** 10.1186/s13002-026-00865-w

**Published:** 2026-03-11

**Authors:** E. Menguy, V. Labeyrie, A. S. Eladine, V. Rafidison, R. J. Randriamalala, D. Renard, S. M. Carrière

**Affiliations:** 1 CIRAD, UMR SENS, F-34398 Montpellier, France; 2 SENS, CIRAD, IRD, UMPV, Univ Montpellier, Montpellier, France; 3 Domaine de Sciences et Technologies, Université de Toliara, Toliara, Madagascar; 4 Faculté des Sciences, Université d’Antananarivo, Mention Biologie et Ecologie Végétales, Antananarivo, Madagascar; 5 Mention Foresterie et Environnement, Ecole Supérieure des Sciences Agronomiques, Université d’Antananarivo, Antananarivo, Madagascar; 6 CEFE, Univ Montpellier, CNRS, EPHE, IRD, Montpellier, France

**Keywords:** Agrobiodiversity, Ethnolinguistic groups, Decision-making, Cultural factors, Socio-economic factors, Semi-arid, Insurance effect, Crop portfolio, Migration

## Abstract

**Background:**

Cultural groups play a key role in shaping and maintaining cultivated diversity, yet their influence is rarely considered in conservation or agricultural development initiatives. However, cultural groups sharing the same territory may hold distinct preferences, knowledge, and practices, which must be acknowledged. Despite recent advances, the processes linking ethnolinguistic identities and cultivated diversity and diversification process remain poorly understood.

**Methods:**

We applied a mixed-methods approach combining a quantitative description of cultivated species frequency and number with a qualitative analysis of 27 semi-structured interviews on farmers motivations to cultivate the different species as well as diversified species assemblage. We compared two ethnolinguistic groups coexisting in the same semi-arid region of Madagascar but with distinct settlement histories, one considered migrant (Antandroy) and the other long-term settled (Masikoro).

**Results:**

We found notable differences in socio-economic conditions and crop portfolio between groups. For some crops, motivations were similar, while others revealed contrasts in cultural and symbolic importance, culinary preferences, and economic traits (e.g., seed access, treatment requirements). Although both groups cultivate a comparable number of species on average, their incentives to diversify differ: Antandroy farmers emphasized spreading food availability through time and the risk-reducing effect of diversity, whereas these motivations were not consistently reported by Masikoro farmers. These patterns reflect intertwined cultural and socio-economic specificities.

**Conclusions:**

Our findings underline the importance of accounting for cultural group differences in crop choices and crop diversity management, even when these groups coexist in the same landscape. Integrating these perspectives is essential for designing more effective conservation and agricultural development initiatives, particularly those supporting crop diversification in risk-prone regions.

## Background

Cultivated plants and their diversity are essential, as they underpin the capacity of crops to adapt to climatic and biological stresses and form the basis of any plant improvement. Yet cultivated plants are not only biological entities, they also bear the imprint of the societies that grow them [1]. This highlights that crop diversity does not arise solely from genetic or environmental factors: kinship rules, alliance networks, and seed selection or exchange practices, for example, shape how plants are transmitted and directly influence their differentiation. All human societies across the world contribute to the evolution of domesticated plants, and to the loss, creation, and maintenance of crop species, varieties, and their diversity in cities [2] and in rural areas [3–10].

Cultivated plants and their diversity are shaped by a wide range of agronomic, ecological, economic, social and cultural factors [11, 78], although the latter are less documented. Notably, indigenous groups are for example often recognized for maintaining particularly high levels of agrobiodiversity [12–15]. Farmers’ ethnolinguistic membership are also known to shape the richness and composition of cultivated plants [16].

Studies show that despite spatial proximity, adjacent cultural groups often cultivate different crops, underscoring the key role of cultural characteristics in shaping cultivated diversity, including species and varietal richness and composition [17, 18]. Similar patterns have been reported for ethnolinguistic groups in other contexts, such as differences in maize morphotypes [19], sorghum morphotypes [20], yam cultivars [21], rice varietal richness [22] as well as species richness and composition in diverse farming systems [23, 24]. Such differences are often attributed to social boundaries regulating seed and information circulation within groups [1, 25, 26]. However, growing research also points to differentiated knowledge, preferences, and values associated with cultivated diversity, which further explain these contrasts. Studies report variation across cultural groups in varietal nomenclature and landrace identification [20, 27–29], as well as distinct trait preferences or values shaping varietal choices [30–32]. Despite these advances, the processes shaping relationships between cultural groups and cultivated diversity remain poorly understood [33].

In addition, cultural differences frequently coincide with economic and demographic differences, for instance concerning land access, household composition and members’ age, farming experience, or wealth. The respective share of culture vs. economic and demographic differences in crop diversity thus remains an open question [28, 32]. In particular, the coexistence of multiple cultural groups within the same area often reflects migration processes, as is frequently the case in the studies cited above, whereby one group has settled in a new environment. A recurrent finding in the literature is that migrant populations, often belonging to different ethnolinguistic groups, tend to cultivate fewer crops than long-established residents, owing to limited knowledge of local environments [13, 34, 35]. Yet Issac et al., show that migrants may maintain broader networks and act as intermediaries, potentially becoming innovation agents by mobilizing external experience [36]. Despite this, studies on crop diversity have traditionally focused on indigenous people and migrants’ perspectives are rarely integrated [37], even though they may contribute to enriching village-level diversity [21]. Greater attention to the perspectives that different cultural groups hold concerning crop diversity is therefore needed.

Yet, cultural groups sharing the same territory may hold distinct aspirations, knowledge, and perceptions of their environment, shaping their practices in different ways [38]. Recognizing and accounting for this linkage between human cultural and crop diversity is therefore essential. Yet, such dimensions remain rarely studied and considered in agricultural development and conservation interventions [31, 35].

The aim of this study is to assess how socio-economic and cultural (ethnicity-related) factors shape crop diversity among farmers from two distinct ethnolinguistic groups coexisting in the same semi-arid landscape. These self-identified groups have contrasting settlement histories, one being considered as migrants to the area, the other identified as more older residents. We use “ethnolinguistic groups” as a cultural identifier based on reported shared origins, including for example history, traditions, practices, knowledge, and linguistic markers [39]. Given potential disparities in socio-economic resources between migrant and previously established households [40], which may confound cultural effects, we first examine whether such differences exist (Objective 1). We then analyse: (i) differences in crop portfolios, considered here as the nature and number of cultivated species (Objective 2), (ii) group-specific motivations for cultivating each species (Objective 3), and (iii) motivations for diversifying crop portfolio (Objective 4). Finally, we integrate these findings to discuss how cultural and socio-economic factors may explain observed crop diversity similarities or differences between groups.

## Methods

### Study area

**Fig. 1:**
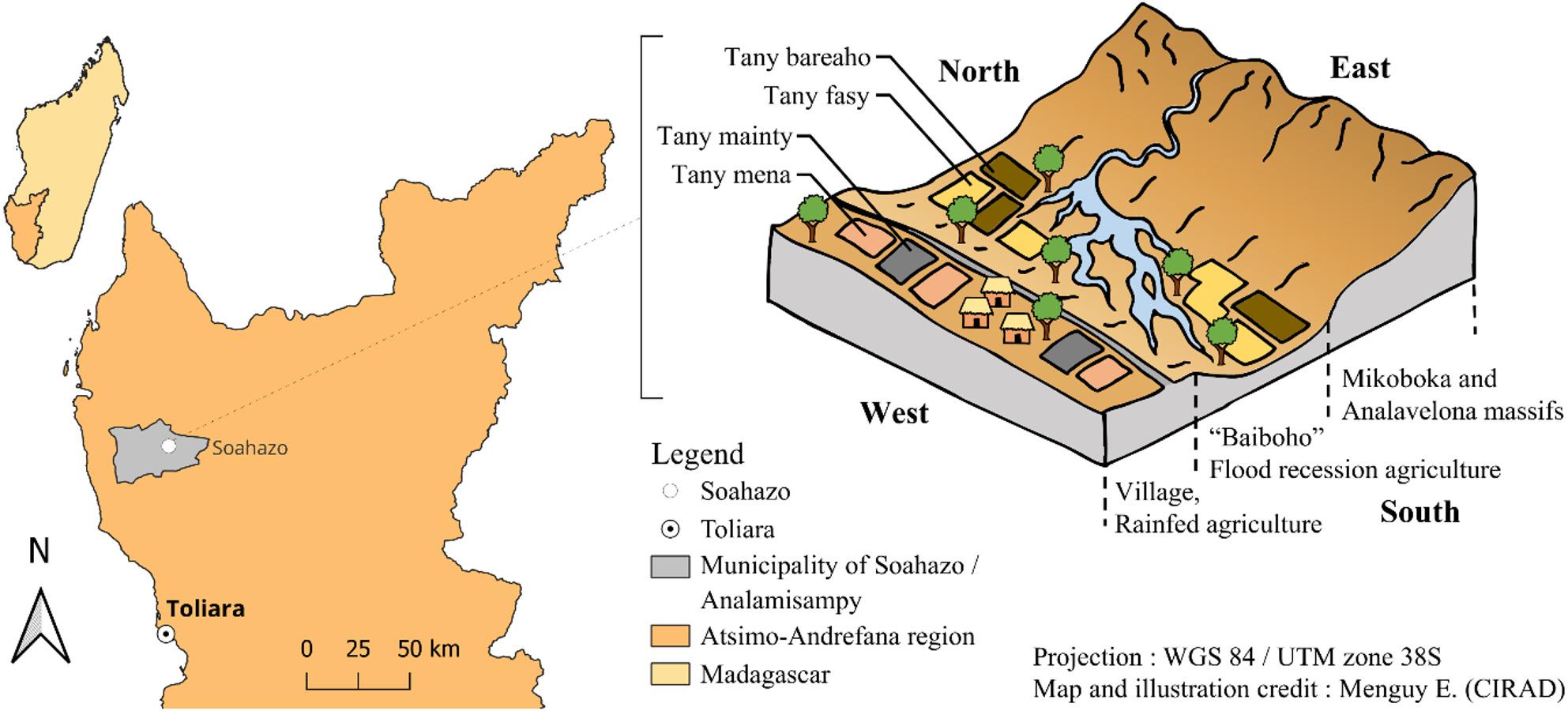
Localisation of the study area in Madagascar and schematic representation of the agricultural landscape. *Tany mainty* = black soil, *tany bareaho* = alluvial soil, *tany mena* = red soil, and *tany fasy* = sandy soil. *Baiboho* = fields in the flood-prone area. Paved road = RN9

The study was conducted in the rural commune of Soahazo, formerly part of the commune of Analamisampy (LAW N°2015-002), located in the Atsimo-Andrefana region in southwestern Madagascar (Fig. 1). The commune of Soahazo had a population of 23,000 inhabitants in 2005 [41], which increased to more than 34,000 inhabitants in 2018 (DATA INSTAT, RGPH-3), reflecting substantial demographic growth. The climate is tropical semi-arid, with average annual temperatures of around 25 °C and average annual rainfall of 800 mm. Soahazo displays a distinctive landscape (Fig. 1), characterized by a low-altitude agricultural plain (200–220 m) interspersed with flood-recession lands known as *baiboho*. East of the RN9, these lands border the flooded hydrographic network of the Tsivora River, which originates in the Mikoboka and Analavelona mountain ranges at more than 1000 m in elevation [42, 43]. *Baiboho* fields are considered the most fertile lands in southwestern Madagascar [44] and led the area to become a hotspot for the commercial cultivation of lima bean between the two World Wars [45], and of cotton from the 1980s onward [46]. In the 1990s, the area also experienced heightened deforestation of the Mikea forest due to slash-and-burn maize cultivation, partly driven by land saturation in *baiboho* fields [42, 47]. At this time, maize shifted from local consumption to a commercial crop, supplying both the national market and exports to Réunion. Following the decline in maize exports and the establishment of the Mikea Forest National Park, most of the cleared lands are no longer cultivated today [48].

**Fig. 2:**
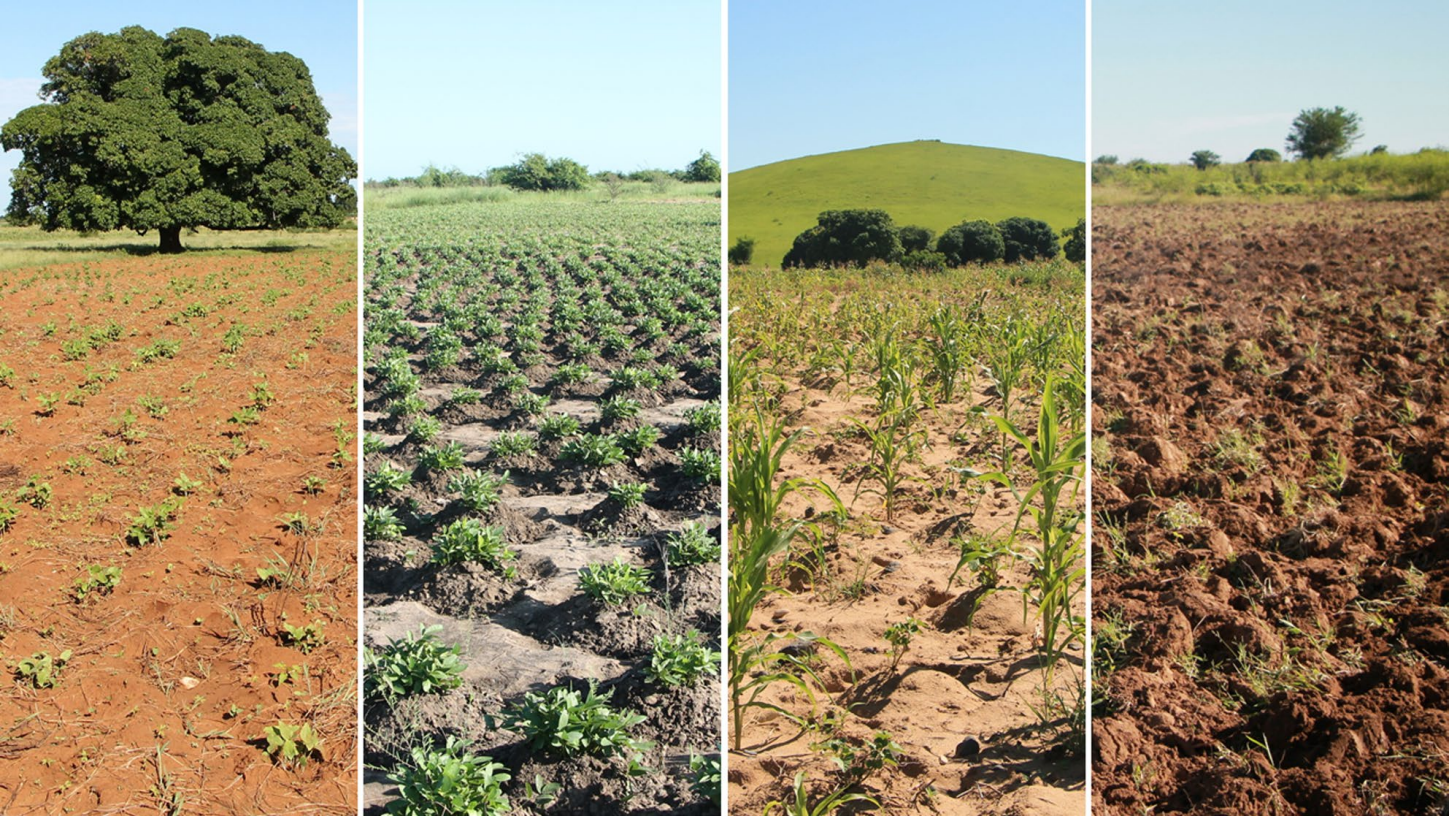
Field photos illustrating dominant soil types in the study area in southwestern Madagascar (left to right): Red soil (*tany mena*) with cowpea (*Vigna unguiculata*); Black soil (*tany mainty*) with Bambara nut (*Vigna subterranea*); Sandy soil (*tany fasy*) with maize (*Zea mays*); Alluvial soil (*tany bareaho*), freshly tilled, ready for lima bean (*Phaseolus lunatus). (© Ewen Menguy*,* 2024)*

Two main agricultural systems currently dominate (Fig. 1): (i) flood recession agriculture in *baiboho* lowlands, also called “fields down below” (*tany ambany* in Malagasy), characterized by alluvial soils (*tany bareaho*) or sandy soils (*tany fasy*) (Fig. 2), which are more or less flood-prone depending on water levels; and (ii) rainfed agriculture on ferruginous soils located on the natural levees near the villages, called “fields up above” (*tany ambany*) [44, 49, 50], composed of red soils (*tany mena*) and black soils (*tany mainty*) (Fig. 2). This pedological diversity allows for the cultivation of various crops, including maize (local name : *tsako; latin name : Zea mays* (L.)), cassava (*balahazo; Manihot esculenta* (Crantz)), sweet potato (*bele; Ipomea batatas* (L.)), cotton (*hasy; Gossypium sp.* (L.)), tobacco *(paraky; Nicotiana tabacum* (L.)), and several legume species: cowpea (*lojy; Vigna unguiculata* (L.)), mung bean (*antsoroko; Vigna radiata* (L.)), lima bean (*kabaro; Phaseolus lunatus* (L.)), rice bean (*antsamby or tsiasisa; Vigna umbellata* (Thunb.)), peanut (*kapiky; Arachis hypogaea* (L.)), lablab bean (*antaky; Lablab purpureus* (L.)) and Bambara nut (*Voanjobory; Vigna subterranean*a (L.)) [51]. Due to the lack of adequate water infrastructures, rice is not cultivated. A weekly market is held every Saturday in Soahazo, attended by collectors from Toliara, and has become more accessible thanks to the recent paving of the RN9.

We conducted our study in two *fokontany* of Soahazo characterized by distinct ethnolinguistic groups, one predominantly Masikoro and the other Antandroy. These two sites were selected for their comparable agroecological and socioeconomic contexts, including similar climatic conditions, soil characteristics, and levels of access to land and local markets.

### Population

#### The *Tompon-tany* Masikoro

The commune of Soahazo is located at the heart of the Masikoro region, the former Antifiherenana kingdown, situated between the Mangoky River to the north, the Onilahy River to the south, and the Analavelona and Mikoboka mountain ranges to the east [52]. Masikoro kingdom, founded 250 years ago, unified under a single political entity a highly mobile people whose main activity was extensive cattle herding [45, 53]. Since then, Masikoro people have been considered *tompon-tany* (literally “masters of the land”), as the first occupants and legitimate landowners of the territory. They established a pact of respect with the spirits of the place and oversee human activities within this territory [53, 54]. *Tanindrazana*, or “the land of the ancestors”, has been transmitted primarily through inheritance to Masikoro descendants, who today cultivate the best lands [45, 55]. Masikoro society is structured around a lineage-based social organization, with cattle herding at the center of their identity and way of life, many traditional ceremonies requiring the ritual sacrifice of a bull, and with most financial gains reinvested in the purchase of zebu cattle [56]. Alongside livestock herding, and encouraged by the settlement of agricultural groups such as Betsileo [52], Masikoro populations progressively developed agriculture in irrigable lands and on the most fertile soils [43, 45], particularly in the Antseva corridor, which includes the commune of Soahazo [44]. The expansion of agricultural land onto the best grazing areas, combined with insecurity linked to zebu theft, gradually reduced cattle numbers and increased the importance of agricultural activities [56, 57].

#### The *Mpiavy* Tandroy 

Because of its low population density, its vast expanses of grazing land, and its agricultural potential, southwestern Madagascar has long been a land of settlement [53], encouraging the arrival of migrants known as *mpiavy* (literally “foreigners”), including Antandroy. Unlike Masikoro, Tandroy people are defined more by their geographical location in the far south of the country, the Androy region, than by a former political unity [43] (Antandroy refers to people living in this area, literally “Those from the land of thorns” commonly known simply as Tandroy people). Tandroy migrations began in the 1930s, after the destruction of the *raketa* (*Opuntia vulgaris*) by a parasitic caterpillar, depriving both people and livestock of a crucial food and water resource during times of crisis [52]. Several migratory waves were thus observed in the southwest. Their departure was first methodically organized by European concessions, which recruited Tandroy people for their “flexibility and relentless capacity for work” [45, 58]. They were later drawn by the cotton boom, a crop that is highly labor-intensive [55, 59]. Market incentives for maize cultivation under slash-and-burn systems also influenced these movements in the Southwest, although this dynamic did not directly concern the study area but rather neighboring zones [47, 58, 60]. Tandroy migrants mainly settled in the former district of Morombe [52], where they accounted for 30% of the population in certain localities, notably Soahazo [43]. Tandroy migrations are rarely permanent: many return to their area of origin in the short or medium term, while those who remain indefinitely maintain enduring ties with their families back home [52]. Tandroys people often settle near families who migrated previously, forming villages or hamlets separate from Masikoro villages and maintaining ways of life that differ from and remain relatively independent of their neighbours [45]. The *fokontany* selected for this study was already recorded as a predominantly Tandroy village in 1967, a rare characteristic according to Dandoy’s atlas [45]. Tandroy migrants generally practice extensive livestock husbandry and accept various types of wage labor, notably as *kibaroa* (task-based workers) carrying out intensive agricultural work often abandoned by the *tompon-tany*. After several years of settlement, Tandroy people seek access to land, whether through loans, sharecropping arrangements, or clearing [43]. Sharecropping contracts, whose traditional forms involved the landowner providing plowing, seeds, and phytosanitary products, while the cultivator carried out all agricultural operations from sowing to harvest, became more widespread after the cotton boom [55].

### Data collection and analysis

Our approach consisted of two steps. The first aimed to assess socio-economic factors and cultivated diversity across the two ethnolinguistic groups. The second examined differences in the motivations for cultivating each species, as well as the diversity of species and varieties grown. Both steps are detailed below.

#### Step 1: Farmers’ socio-economic factors and crop diversity assessment

For the first step, direct field observations were conducted during the 2024 rainy season to document species richness and crop portfolio, that is, the set of crop species cultivated within each household [61, 62]. We interviewed 37 farmers of whom 22 identified as Masikoro farmers and 15 as Tandroy farmers. Farmers were surveyed opportunistically as we encountered farmers in their fields, while ensuring that the full range of crop portfolio present in the region was represented. We visited 70 fields out of a total of 106 (66.0%) managed fields by the 37 farmers. Age and gender, being influential factors in cultivated diversity [32, 35, 63], were also balanced across groups to ensure comparability. We aimed to visit all cultivated fields for each farmer, thought about one third of the fields were inaccessible, unsown, not yet obtained for sharecropping or not directly cultivated by the farmer (given to someone for sharecropping). During each visit, we recorded the main crop species grown in the field (excluding plants cultivated in the edge of the field such as cucurbits, watermelon, tomatoes and sometimes lablab bean) and basic field characteristics (estimated area, ownership or sharecropping status, and flood-recession vs. rainfed conditions). We did not include owned plots that were not cultivated by the farmer during field visits. We also collected information on the farmer (age, sex, ethnolinguistic group, number of draught zebu) and, when necessary, completed the list of species cultivated in unvisited fields. By compiling these observations, we constructed a farm-level database indicating the presence or absence of each cultivated species, complemented by socio-economic variables. These included the number of draught zebus as a proxy for plowing equipment, as well as the total number and estimated area of cultivated fields, along with the proportion of these fields that were owned or located in flood-recession zones. Such variables are commonly used as local wealth indicators in the area [48, 57]. Given that flood-recession fields are less accessible for purchase or sharecropping due to strong demand [42, 44], we added three binary variables (yes/no): cultivation of at least one flood-recession field, ownership of at least one field, and ownership of at least one flood-recession field.

All analyses were conducted in R (version 4.3.2). We first used Wilcoxon signed-rank tests for quantitative variables and Fisher’s exact tests for binary variables to assess whether socio-economic characteristics differed between ethnolinguistic groups. To compare farmers’ crop portfolio among ethnolinguistic groups, we used the species inventory to generate frequency histograms. We tested for differences in species frequency between ethnolinguistics groups using Fisher’s exact test and compared the average number of species cultivated per farmer using the Wilcoxon signed-rank test. Using the “vegan” package in R [64], we conducted a Principal Coordinates Analysis (PCoA) to represent farmers’ species portfolio based on the species inventory. This matrix allowed us to compute distances between farmers based on their cultivated species: the more similar the portfolio, the shorter the distance. The distance between farmers was measured using the Jaccard dissimilarity index, the common distance measurement for presence/absence data [65]. To assess whether ethnolinguistic group explained variation in farmers’ species portfolio in the two-dimensional PCoA, we used a permutation test [66], comparing the observed correlation to those from 999 random permutations of the data.

#### Step 2: Crop diversity choice assessment 

For the second step, farmers’ motivations for cultivating the different species as well as diversified species portfolio were documented through 27 semi-structured interviews conducted with 14 Tandroy farmers and 13 Masikoro farmers. Farmers were selected to capture a gradient of species richness in cultivated species. Among these participants, 21 were involved in both field observations and interviews.

The interviews, which lasted approximately two hours on average, consisted of three main parts. In the first part, we began by reviewing all cultivated and non-cultivated fields by the farmer. When relevant, we asked farmers about their motivations for not cultivating flood-recession fields and the terms of sharecropping arrangements. We also explored farmers’ motivations for not cultivating some of their owned fields (not observed during field visits). The second part focused on the motivations that encourage or limit the cultivation of each species. We used a list of the main species cultivated in the area and a straightforward question: “What makes this species important or unimportant to you?” We collected farmers’ responses categorizing them as advantageous or disadvantageous. The third part of the interview explored the motivations that encourage the cultivation of diverse species and varieties. For each cultivated species, we asked farmers to report the number of distinct varieties they cultivated, considering varieties as named and reported by the farmers [67]. Based on the assemblage species and varieties grown, farmers were asked, “What led you to grow multiple species or varieties?”. Based on participants’ responses, several follow-up questions were posed to further understand their choices. The interviews were carried out in French by the first author (E.M.) and translated in the local language by the third author (E.A.S., Malagasy speaker). With the consent of the participants, the interviews were recorded and subsequently transcribed. We compared the average number of varieties between ethnolinguistics groups using a Wilcoxon signed-rank test. Those analysis were performed only for species with intraspecific diversity (maize, cowpea and sweet potato). 

Analysis of part 1. was conducted by compiling respondents’ answers regarding land issues, identifying farmers concerned and examining the reported problems. For part 2. we conducted a deductive analysis by coding farmers’ motivations using the framework proposed by Demongeot et al. [78], which classifies the “values” associated with cultivated species into six domains: cultural symbolic meaning (e.g., heritage, intangible forces), cultural preferences (e.g., culinary or storage qualities), socio-economic (e.g., seed access, labor, cash income, food self-sufficiency), ecological interactions (e.g., microclimate, soil fertility), maintenance of options, and agroecological traits (e.g., cycle, input requirements). Each motivation was coded by species, thematic category (level 2 from Demongeot et al. [78]), and whether it was perceived as an advantage or a disadvantage. When multiple motivations fell within the same category for a given species and respondent, they were grouped. We then examined, within each of the six domains, similarities and differences between ethnolinguistic groups and the crops concerned, highlighting categories emphasized by one group more than the other or cited for specific crops only.

Analysis of part 3 was built on previous work [51], where full methodological details are provided. In brief, we conducted a thematic analysis, using interview transcripts and NVivo 14. We generated inductively 9 main themes reflecting the diversity of motivations reported by farmers for cultivating species and varietal diversity. For this article, we re-examined farmers’ accounts using the same thematic structure, but considering discourse as constitutive rather than merely reflective of personal realities, allowing us to explore how meanings are constructed in relation to respondents’ ethnolinguistic group [68]. In this article, we focus only on the themes where clear differences between ethnolinguistic groups were identified.

## Results

### Socio-economics characteristics varies between ethnolinguistic groups

Our results reveal socio-economic asymmetries between ethnolinguistic groups in access to flood-recession fields. Although quantitative comparison shows no significant differences in the average number or area of cultivated, owned, or flood-recession fields, nor in plowing equipment (Table 1), a significantly smaller proportion of Tandroy farmers cultivated at least one flood-recession field (Fisher’s exact test, *p* = 0.03057). Importantly, however, they still cultivate owned plots at comparable levels to Masikoro farmers, indicating that the difference concerns access to this specific land type rather than land cultivation in general. Only 1/22 Masikoro farmer do not cultivate such fields, compared with 5/15 Tandroy farmers. Conversely, seven of the 15 Tandroy farmers cultivated more than 2 ha of flood-recession land, compared with five of 22 Masikoro. This pattern highlights asymmetries both between groups and within Tandroy community, where some farmers lack access while others manage substantial areas. Qualitative insights suggest that differences between ethnolinguistics groups relate to unequal financial resources, as illustrated by a farmer seeking a sharecropping agreement in a flood-recession area who explained:

*“I tried*,* but I didn’t get one. It’s often those who have zebus who are given land like that […] because they know you’ll do the ploughing yourself…”* (Farmer 1, Antandroy).

While ploughing and other costs are normally borne by the landowners, traditional sharecropping arrangements appear to be shifting under increasing land pressure.

*“Landowners feel entitled to ask for many things because everyone wants to cultivate* [on flood-recession fields]* […] they require the cultivators to plough*,* plant*,* and even provide the seeds themselves. Meanwhile*,* the landowner benefits without having spent anything”* (Farmer 12, Antandroy).

Such dynamics are not limited to flood-recession fields and were also reported by two Tandroy farmers who cultivate fewer rainfed fields than desired due to unfavorable sharecropping terms. Masikoro farmers face similar constraints, but five reported continuing to cultivate these fields despite having to purchase seeds or other inputs, a situation not mentioned by any Tandroy farmer.

Uncultivated fields also reflect financial disparities between ethnolinguistic groups. Although similar numbers of Masikoro (5) and Tandroy (6) farmers leave fields uncultivated, the reasons for doing so differ markedly. Among Masikoro farmers, leaving fields fallow reflects financial limits (for ploughing, seeds) within otherwise extensive operations, rather than acute vulnerability. In contrast, among Tandroy farmers, only one faced this same constraint. The others abandoned their own fields entirely due to lack of means and were forced to find traditional sharecropping arrangements, where landowners cover input costs.

*“I no longer farm my own field because I have no funds*,* so I take a sharecropping contract where the owner provides everything”* (Farmer 6; Antandroy).

Similarly, one Tandroy farmer with limited resources used evolving sharecropping arrangements as a way to access seeds:

*“Other sharecroppers had seeds*,* so I told them to farm my own field while I farm elsewhere where the owner provides seeds”* (Farmer 21, Antandroy).

**Table 1:** Summary of respondents’ socioeconomic characteristics, presented as means or percentages by ethnolinguistic group

	Antandroy	Masikoro	Total*	*p*-value
**Sample size**				
No. of individuals	15	22	37	-
**Gender (%)**				
Women	33.33	31.82	32.43	-
Men	66.67	68.18	67.57	-
**Age (%)**				
18–35 years	33.33	36.36	35.14	-
35–50 years	33.33	40.91	37.84	-
+ 50 years	33.33	22.73	27.03	-
**No. of draught zebus for ploughing (%)**				
No zebu	60	68.18	64.86	NS
1 zebu	13.33	4.55	8.11	NS
2 zebus	26.67	18.18	21.62	NS
4 zebus	0	9.09	5.41	NS
**No. of fields**				
Average number of fields	2.73	2.95	2.86	NS
↳ Including flood-recession fields	1.33	1.55	1.46	NS
↳ Including owned fields	1.6	1.59	1.59	NS
**Surface (ha)**				
Average total field area	3.2	3.14	3.16	NS
↳ Including flood-recession area	1.59	1.33	1.44	NS
↳ Including owned area	1.78	2	1.91	NS
Fiels acces and ownership (%)				
Farmers cultivating ≥ 1 flood-recession field	66.7	95.5	83.8	*
Farmers owning ≥ 1 field	86.7	77.3	81.1	NS
Farmers owning ≥ 1 flood-recession field	26.7	36.4	32.4	NS

* The “Total” column shows values for all respondents combined

### Crop diversity varies between ethnolinguistic groups

**Fig. 3:**
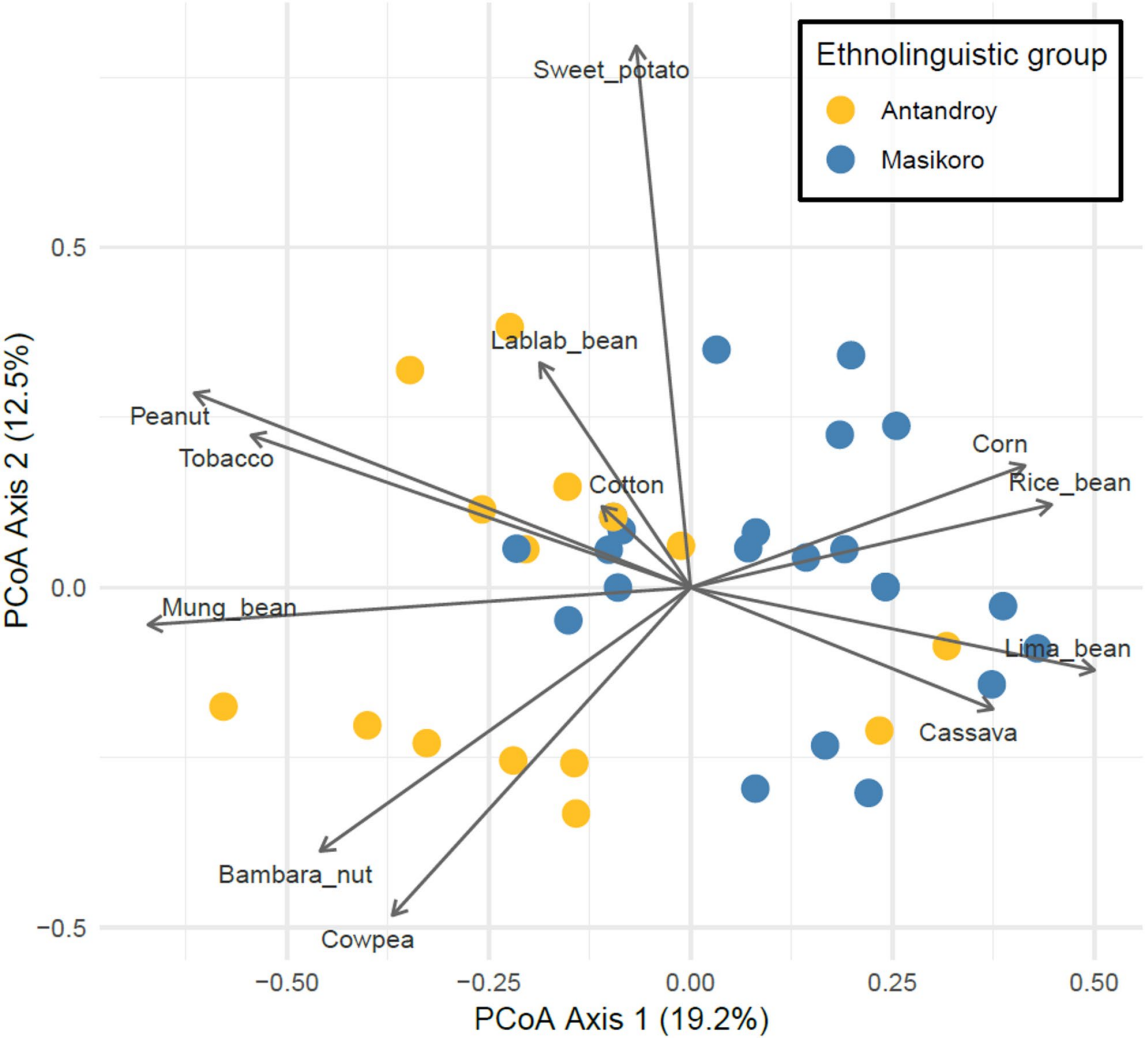
Principal Coordinate Analysis (PCoA) of cultivated species based on Jaccard distance. Points represent farmers, colored by ethnolinguistic group. Percentages in parentheses indicate the proportion of variance explained by each axis

Although species richness does not vary between ethnolinguistic groups, the species portfolio differs between them. On average, farmers cultivated 4.81 species (SD = 2.05), with no significant difference between ethnolinguistic groups (Masikoro: M = 4.82, SD = 1.90; Antandroy: M = 4.82, SD = 2.20). Figure 3 illustrates the distribution of farmers based on the species they grow. Ethnolinguistic groups membership significantly influences species portfolio composition (permutation test, R²=0.2166, *p* = 0.001). Farmers cultivating a wide diversity of species (center of the plot) stand apart from more specialized groups. These specialized groups cluster according to their dominant crops: cassava, or rice bean for Masikoro (right of the plot) and lablab bean, peanut and tobacco (top left) or mung bean, cowpea and Bambara nut (bottom left) for Tandroy farmers.

**Fig. 4:**
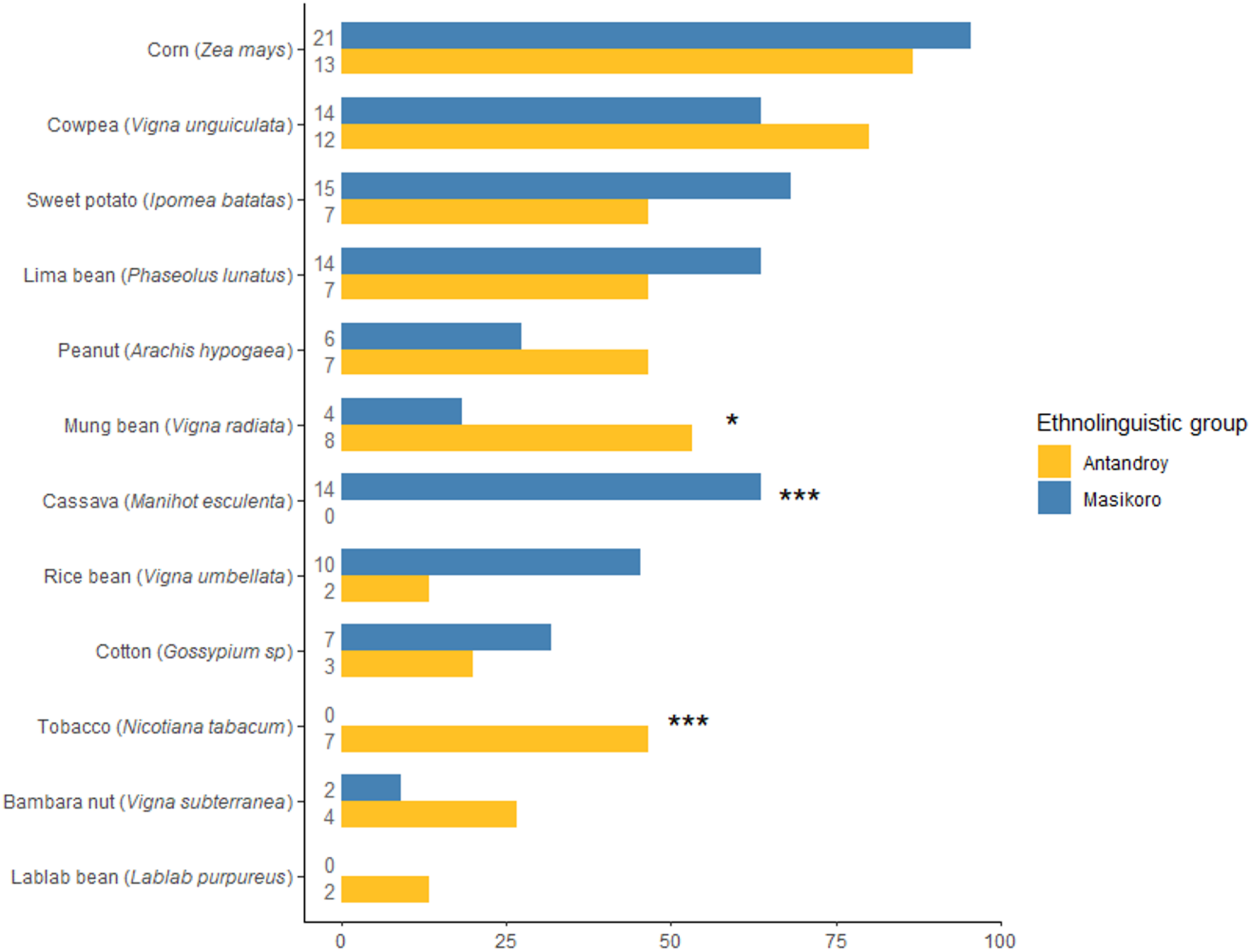
Cultivated species by ethnolinguistic group (% of farmers growing each species). Asterisks indicate significant differences between groups (Fisher’s exact test) and numbers on the left of the bars show the number of farmers in each ethnolinguistic group who grow the species

Although Masikoro and Tandroy farmers share many species in common, variation in the cultivation frequency of specific crop was observed between ethnolinguistic groups (Fig. 4). Mung bean is significantly more commonly grown by Tandroy farmers than by Masikoro farmers (Fisher’s exact test, *p* = 0.036). Cassava is grown exclusively by Masikoro farmers (Fisher’s exact test, *p* = 0.00007), while tobacco (Fisher’s exact test, *p* = 0.00063) and lablab bean (non-significant, Fisher’s exact test, *p* = 0.158) are grown only by Tandroy farmers.

At the varietal level, the mean number of varieties cultivated per farmers does not differ significantly according to ethnolinguistic groups for species exhibiting intra-specific diversity: maize, cowpea and sweet potato. The only difference lies in number and management of lablab bean varieties: Tandroy farmers grow one to three distinct varieties along field borders, while Masikoro farmers grow only one. Tandroy farmers also allocate larger plots to a single dominant variety.

### Crop reported advantages or disadvantages vary between ethnolinguistic groups

While both groups attributed similar advantages and disadvantages to cultivated species, some differences emerged. Each ethnolinguistic group associated distinct cultural and symbolic values with specific crops as well as distinct culinary and taste preference and income generation motivations. Also, Tandroy farmers mainly emphasized seed access, land availability, plant protection needs, and the possibility of multiple harvests while Masikoro farmers reported price stability and labor requirements as key considerations. See Table 2 for a species-by-species summary of the perceived advantages and disadvantages reported by both groups.

#### Cultural and symbolic meanings

Farmers reported some crops as culturally associated to Masikoro or Tandroy farmers, while others were valued by both.

Farmers from both ethnolinguistic groups linked maize, cassava, cowpea and lima bean to heritage and collective identity, frequently described under the notion of *faharaza* (literally “since the time of the ancestors”) and rooted in long-term cultivation, as stated:

*“Maize and cassava are our ancestor’s crop*,* so whatever we grow*,* those two are always part [of our portfolio].”* (Farmer 10; Masikoro);

“*Here we cannot grow rice […] we only grow maize*,* cassava*,* lima bean*,* this is our culture*” (Farmer 2, Antandroy).

Farmers also reported a recent revival of lima bean cultivation, reflecting a desire to restore crops perceived as culturally embedded components of the local landscape:

“*Lima beans*,* that’s what we cultivate everywhere here… it was the culture of the ancestors. But at some point*,* it stopped working. But some tried it again a few years ago and it worked well. So*,* since then*,* everyone has started growing it again*,* because it is our traditional crop*,* and everyone is returning to the old culture*,* and everyone wants to grow lablab beans again like before*” (Farmer 27, Masikoro).

But its past decline had favored alternatives crops such as rice bean. Today, only Masikoro farmers expressed strong attachment to rice bean, rooted in habit and long-term cultivation:

“*It’s my crop*,* even if I wanted to quit*,* I couldn’t […] it is our ancient crop”* (Farmer 8, Masikoro).

In contrast, only Tandroy farmers associate Bambara nut, peanut, tobacco, and lablab bean with heritage and collective identity. Masikoro farmers acknowledged these cultural links between those crops and Tandroy farmers as well and sometimes reported lacking the required knowledge to grow certain of this crop such as tobacco.

*“Lablab has been passed down from our people. I kept the seeds […] That’s why it’s always in our fields. We can’t do without it*,* it comes from our ancestors*,* from my father”* (Farmer 21, Antandroy).

“*As for tobacco*,* the story is that first of all*,* it’s people who come from Androy*,* who come from the south. And when they arrive*,* they find soils that are good for tobacco*,* for peanut*,* for Bambara nut*,* and then they cultivate them here. But these crops come from their region*” (Farmer 22, Masikoro).

Bambara nut cultivation is also considered *fady* (taboo) by some Masikoro farmers, an inherited prohibition whose original rationale is no longer clearly known. Individuals from both groups wearing protective zebu-theft talismans (called *fiaro* or *mohara*) also avoid consuming or cultivating it, as it is believed to diminish their effectiveness.

#### Cultural preferences

Some difference in culinary and taste preferences also emerged between both ethnolinguistic groups.

Both groups highlighted the taste qualities of cowpea and lima bean and their use as side dishes. They also emphasized the long-term storability of cassava and sweet potato, particularly when the latter is processed into *piky* (dried slices). In contrast, only Tandroy farmers reported that peanut have nutritive qualities substituting for oil in the diet. Only Masikoro farmers grow lablab bean along field margins and consume it fresh, while some Tandroy farmer also grow it in open fields and dry it for long term storage. These differences in terms of cultivated surface and motivations to eat highlight a contrasted role of this crop in the subsistence of Masikoro and Tandroy farmers, as a Masikoro farmer described:

*“Here we eat lablab fresh*,* with cassava. We don’t eat it dry. […] It’s always been a minor crop*,* complementing the major ones. […] In Androy*,* they took it seriously because there wasn’t enough water. Lablab thrives under drought. […] For them*,* it became a staple food”* (Farmer 24, Masikoro).

Nevertheless, despite the importance of this crop for them, some Tandroy farmers lacking land rights and plant only small amounts along field margins:

*“Most landowners here do not allow lablab cultivation*,* so we just use the field edges to plant a few”* (Farmer 18, Antandroy).

#### Socio-economic and agroecological traits

Socio-economic considerations and agronomic traits were closely intertwined in farmers’ reports, for example, production costs were largely attributed to weeding and phytosanitary requirements, so they are presented together here.

Both groups shared several general economic considerations regarding their cultivated plants. Farmers from both groups agreed that maize requires little labour and expenditure but provides low income, while cotton is the more costly and labour-intensive yet offers the highest returns. Cowpea, mung bean, rice bean, and lima bean were also perceived as costly due to high pesticide needs, but highly profitable as cash crops. Maize, cassava, and sweet potato were emphasized for household food supply, and both groups highlighted the short cycles of maize and cowpea for providing early food and seasonal income:

“*Because it is harvested very early and it will save us from famine*” (Farmer 16, Antandroy).

The only notable disparity concerned mung bean: only Tandroy farmers stressed its early provision, whereas Masikoro farmers emphasized its high yields.

Tandroy farmers mainly emphasized seed access, land availability, plant protection needs, and the potential for multiple harvests. Constraints in access to planting material were mentioned only by Tandroy farmers for peanut, rice bean and lablab bean. However, limited access to cassava cuttings, due to their high sensitivity to cyclone-induced flooding and prolonged drought, and the high cost of cowpea and lima bean seeds were reported by both groups. A farmer explained:

*“Here*,* it has become very difficult to obtain cassava cuttings. And it got worse this year because there were* tagnanandro (literally “sunburn”, generally coming from delays in rainfall) *which caused all the cassava cuttings to dry out”* (Farmer 13, Antandroy).

While low treatment requirements were noted by both groups for maize, peanut, and Bambara nut, only Tandroy farmers mentioned similarly low treatment needs for sweet potato and cassava, as well as the high requirement for cotton.

“*When I farm*,* it’s always maize*,* cassava*,* and sweet potato*,* because cassava and sweet potato don’t need any pesticide at all*,* and maize just needs a little pesticide with some ash*,* and it works*” (Farmer 2, Antandroy).

They were also the only ones to highlight the ability of tobacco and cowpea to be harvested multiple times. Only tandroy farmers reported land access as a constraint and particularly emphasized challenges in securing crop recession fields for crops such as lima bean:

“*Plots of land I find are not suitable for lima bean*,* so I grow sweet potato*” (Farmer 21, Antandroy).

Conversely, they take advantage of tobacco, which can be cultivated on small plots within the village without requiring access to larger external fields.

Masikoro farmers, by contrast, mainly highlighted price stability and labor requirements as key concerns. Tobacco is a good example illustrating the different income generation strategies among both groups. While Antandroy farmers value it for its regular income potential, Masikoro farmers, viewed it as less attractive due to slow, incremental sales as farmers explained:

“*Masikoro farmers can’t understand that tobacco makes a lot of money. Why not? Because tobacco isn’t sold all at once*” (Farmer 6, Antandroy);

“*You need something that sells quickly to get your money. Tobacco sells little by little*,* you have to wait. We need to grow something that brings in a lot of money at once”* (Farmer 24, Masikoro).

Additionally, only Masikoro farmers emphasized price stability concerns for lima bean and coton as illustrated by a farmer:

“*Lima bean sells for around 1000 Ariary per cup*,* and even when the price drops*,* it’s still 700. […] But sweet potato prices collapse. I have some now*,* but I wonder if I’ll find buyers. Lima bean prices are much more stable”* (Farmer 13, Masikoro).

Finally, while both groups agreed that tobacco and Bambara nut are difficult to cultivate, Masikoro farmers were particularly insistent on labor-related constraints. They emphasized the small seed size and daily field presence required for mung bean harvest, as well as the labor-intensive thinning operations needed for cotton.

**Table 2:** Advantages (Ad) and drawbacks (Db) of the 12 main cultivated species in Soahazo (southwestern Madagascar), distinguishing shared and ethnolinguistic group–specific farmer reports

Photo	Species	Shared advantages and drawbacks	Specific advantages and drawbacks	
			Masikoro	Antandroy
	Maize *(Zea mays)*	Ad: Cultural, main staple food, low-cost crop (low treatment needs), low-effort crop (minimal weeding), short cycle, early food/income, surplus for sale Db: Low selling price, poor grain storage (pests)	Ad: Two annual harvests (rainy and dry season)	Ad: Yield stability
	Cowpea *(Vigna unguiculata)*	Ad: Cultural, short cycle, early food/income, side dishes, good taste, high income Db: High production cost (seed + treatments), late grain germination risk (due to rain)	—	Ad: Multiple harvests
	Sweet potato *(Ipomea batatas)*	Ad: Food self-sufficiency, long-term storability (when dried in *piky*), staple food with surplus for sale Db: Needs sandy soil, unstable prices	Ad: Yield stability	Ad: Low treatment needs
	Lima bean *(Phaseolus lunatus)*	Ad: Cultural, adapted to *bareaho* soil, side dishes with good taste, high income Db: High production cost (seed + treatments)	Ad: Stable price	Db: Limited access to suitable land
	Peanut *(Arachis hypogaea)*	Ad: Low treatment needs, high market value	—	Ad: Cultural, can replace oil in diets Db: High seed cost
	Mung bean *(Vigna radiata)*	Ad: High income, short cycle, occasional side dish Db: High production cost (treatment)	Ad: High yield Db: Small seeds, daily field presence for harvest	Ad: Early food/income
	Cassava *(Manihot esculenta)*	Ad: Cultural, main staple food, long-term storability, nutritious Db: Climate affects cutting access	—	Ad: Low treatment needs
	Rice bean *(Vigna umbellate)*	Ad: High income Db: High production cost (treatment)	Ad: Habits (adopted to replace lima bean), good market sales	Db: High seed cost
	Cotton *(Gossypium sp.)*	Ad: Most lucrative crop Db: High labour/treatment cost, company inputs require repayment	Ad: Stable price Db: Labour-intensive tasks (e.g. thinning)	Db: Non-consumable, high treatment needs
	Lablab bean *(Lablab purpureus)*	Ad: Drought-tolerant	Ad: Side dish when consumed fresh Db: Limited market demand	Ad: Cultural, dried for storage, staple food (dried) and side dishes (fresh) Db: Difficult seed access, long cycle
	Bambara nut *(Vigna subterranea)*	Ad: Low treatment needs Db: Labour-intensive ridging, avoided by those with zebu theft talismans	Db: Considered *fady* (taboo) by some	Ad: Cultural, food + income
	Tobacco *(Nicotiana tabacum)*	Db: Most labour-intensive crop, needs watering/shade	Db: Limited cultivation knowledge, low income	Ad: Cultural, regular income, early and multiple harvests, low-labour periods

### Motivations for crop diversity vary between ethnolinguistic groups

Motivations for cultivating crop diversity differed between ethnolinguistic groups: Tandroy farmers mainly emphasized insurance and food-related motivations, whereas only Masikoro farmers highlighted economic benefits. While Masikoro farmers also pointed to human capital constraints and strong interest in yield potential as limiting factors, Tandroy farmers mentioned various ones but without a dominant theme emerging from comparison.

About half of Tandroy respondents mentioned seeking insurance against risk during interviews, while only a minority of Masikoro respondents did so. For Tandroy farmers, insurance motivations were central in justifying the cultivation of multiple species, as illustrated here:

*“If there is a lot of rain*,* some [species] will resist because they like it*,* and if there is not enough rain*,* others will like that and manage. For example*,* cowpea needs rain that comes today and then a little more after a few days*,* but not rain that keeps falling all the time. Bambara nut*,* on the other hand*,* need constant rain*,* they need a lot of water*,* and even if they’re flooded*,* it’s good for them. So I grow many [species] so that if there’s a lot of rain*,* some will resist and thrive; if there’s not enough rain*,* others will thrive and survive”* (Farmer 5, Antandroy).

While both groups mentioned economic motivations, only Tandroy farmers explicitly associated risk mitigation with this dimension, particularly with inheritance and ancestral transmission, as illustrated by the following proverb:

*“*Torobory sankena - *it’s a Tandroy proverb that says you should aim for birds that have meat. […] If you’re used to choosing birds that have meat*,* then even if you miss and don’t get one today*,* at least when you go home*,* you still have the meat you got yesterday. […]. So when we farm*,* we think about what we can grow that will help us in times of risk. When I grow many things*,* I know that if one fails*,* others will succeed. Because I’ve planted several*,* not everything will be destroyed at the same time”* (Farmer 18, Antandroy).

Similarly, about half of Tandroy farmers referred to food-related motivations at both the species and varietal levels, while only a minority of Masikoro did so. At the species level, Tandroy farmers emphasized food security:

“*What I grow doesn’t bring much profit*,* but it’s what keeps my children alive. I have many children*,* and for them to eat*,* there must always be something. It’s what I harvest that feeds them”* (Farmer 4, Antandroy).

At the varietal level, they highlighted the advantage of staggering harvests by combining varieties with different growth cycles, as illustrated by this farmer:

“*The benefit of having two varieties is that the first one matures early*,* allowing us to start consuming it. The other*,* with a longer cycle*,* ensures that we continue to have fresh maize. So*,* each time*,* I can harvest and eat again”* (Farmer 19, Antandroy).

While Tandroy farmers place major emphasis on food security, only Masikoro farmers highlighted that cultivating multiple species aims to increase their total income as illustrated by the following quotes:

*“It’s the fact of having a lot and selling a lot to have a lot of money that pushes me to cultivate a lot”* (Farmer 8, Masikoro).

In contrast, only Masikoro farmers are highlighting human capital constraints as limiting factors for species diversification. In particular, they reported the increased labor time and physical effort required to manage a diverse cropping system:

*“It would be difficult to manage everything. With cotton*,* by the first week*,* once it germinates*,* it needs thinning. Otherwise*,* it gets ruined. So that’s where the work for cotton begins. And if I also want to grow mung bean and cowpea*,* it would complicate things. But growing just one of the two along with cotton makes the work more manageable”* (Farmer 27, Masikoro).

At the varietal level, the preference for a single variety per species often reflects a dominant specific attribute. While both groups were seeking short-cycle varieties, only Tandroy farmers emphasized low input requirements in their selection criteria, whereas only Masikoro farmers highlighted high yield potential, as illustrated by one respondent:

*“This is the only one I like because its yields are significant”* (Farmer 22, Masikoro).

## Discussion

This study aims to understand the differences in crop diversity and associated motivations between two ethnolinguistic groups sharing the same landscape. We show the combined influence of socio-economic (linked to migratory status) and cultural characteristics specific to each group on cultivated diversity. We first discuss the similarities between the groups, followed by the divergences attributable to unequal access to resources and to differentiated cultural representations of labor, the temporality of needs, and risk. Finally, we outline the limitations and future perspectives of this research.

### Similarities in socio-economic characteristics and cultivated diversity

At the socio-economic level, our results reveal few differences between the ethnolinguistic groups in terms of agricultural equipment, number of fields, or cultivated area (whether through sharecropping or ownership). However, our study indicates an evolution in land tenure relations: although the status of *tompon-tany* was historically reserved for Masikoro people [45], Tandroy migrants who have been settled for several decades, the earliest Tandroy families having arrived in the area before 1967 [45], appear to have gradually gained access to land ownership, including over alluvial lands. These changes seem to be shaped by social relations, notably marital alliances between ethnolinguistic groups [69] or by the replacement of Masikoro lineage-based land rights with the official legal framework, which promotes individual land registration and thereby weakens Masikoro territorial control [45, 54]. Unlike other regional contexts (e.g., Menabe), Tandroy farmers in the study area have contributed little to slash-and-burn deforestation [47], which has limited interethnic tensions around land use and may also help explain their gradual acquisition of agricultural land [53]. Regardless of ethnolinguistic affiliation, strong disparities persist in access to resources, only one-third of farmers own zebus or alluvial fields, confirming inequalities already documented elsewhere [57].

In terms of the types of cultivated species, with the exception of a few crops such as tobacco or cassava (to which we return later), most species are shared by the two ethnolinguistic groups, suggesting the existence of exchanges and connections among farmers. Indeed, Tandroy farmers have adopted certain Masikoro practices, such as the cultivation of lima bean in flood-recession areas, an activity to which some now attribute an identity value, as our results illustrate. Conversely, Masikoro farmers cultivate species that Tandroy farmers consider as inherited (e.g., peanut, Bambara nut, lablab bean), although they do not ascribe the same value to them. Voorhaar et al. showed that minority groups continue to cultivate plants culturally associated with their identity, independently of the environment [31]. Raimond et al. showed that in addition to growing plants obtained in their host region from local farmers, migrant farmers also cultivate plants brought back during regular returns to their regions of origin [70]. As observed elsewhere [17, 21], it is therefore likely that some seeds accompanied, and may still accompany, Tandroy migration before being integrated locally, as illustrated by this testimony:

“*Now we mix everywhere*,* so even the crops mix*” (Farmer 12, Antandroy).

This phenomenon of hybridization of cultivated plants is not isolated and has already been documented in other regional contexts. For example, in the lower Mangoky region (~ 100 km north of Soahazo) Tandroy migrants have become demographically more numerous in some villages, prompting Masikoro residents to adopt Tandroy social and agricultural practices. In contrast, near the Mangoky delta, some Tandroy communities are so deeply integrated that they now identify as Masikoro [49]. Despite these varying degrees of integration, both groups frequently exchange crops and agricultural techniques, as illustrated by the widespread cultivation of rice, introduced by Betsileo migrants from Madagascar’s highlands. Globally, migration shapes which crops people grow, not by erasing cultural preferences, but by mixing them. Depending on how open the host society is and how strongly migrants hold onto their traditions, they may keep, replace, or add new species to their fields [71]. In many cases, this blending doesn’t dilute identity, it enriches local crop diversity, turning migration into a source of agricultural resilience.

Regarding cultivated-plant diversity, our results show that the two groups grow a comparable richness of species and varieties, suggesting that migrant farmers maintain levels of crop diversity similar to those of local groups. This contrasts with the common assumption that local communities safeguard higher diversity [12, 13, 34]. This pattern may reflect hybridization processes or the fact that migrant farmers draw on a diverse set of seed sources, including seeds from their places of origin, which together generate highly diversified species and variety portfolio [70].

### Socio-economic differences and cultivated diversity

Despite broadly similar production conditions, our study highlights notable differences between the ethnolinguistic groups, linked in part to unequal access to land, economic, and social resources that can influence cultivated diversity. Our results show that a smaller proportion of Tandroy farmers cultivate at least one alluvial field (*baiboho*). This land inequality appears to directly shape crop diversity: crops such as lima bean and rice bean, which are well adapted to these soil types, are more prevalent for Masikoro farmers. Tandroy farmers, who emphasized the difficulty of accessing land suitable for lima bean cultivation, likely orient their choices toward species adapted to fields cultivated during the rainy season, such as mung bean, a non-inherited crop but one grown more widely by Tandroy than by Masikoro farmers.

Moreover, the land disparities observed reveal financial inequalities between the two ethnolinguistic groups, which are also reflected in the advantages or constraints associated with the cultivated species. Tandroy farmers more frequently emphasize aspects related to the purchase of phytosanitary products, and they insist more than Masikoro farmers on the low treatment requirements of species such as peanut, maize, sweet potato, or Bambara nut. The lack of liquidity, already documented among different ethnolinguistic groups [40] and in other contexts within our study area [44], emerges as a key factor structuring species portfolio.

The combined analysis of cultivated species and the advantages or constraints associated with them can also reveal asymmetries in access to seeds. The case of cassava is particularly telling: all farmers reported a decline in production due to droughts and cyclones since Cyclone Haruna in 2013, making access to cuttings a major issue in the area. As one Masikoro farmer noted:

“*It was after that that we started having problems finding cuttings*,* but before that*,* everyone grew cassava*” (Farmer 27, Masikoro).

In contrast, Tandroy appear more affected than Masikoro farmers, as none of them currently cultivate cassava despite a similar perception of its advantages (e.g., hardiness, contribution to food self-sufficiency). This suggests more restricted access for migrants to local seed-exchange networks [25] potentially reinforcing structural inequalities between groups, as illustrated by this testimony:

“*I already know that the people from whom I could ask for cuttings do not have any. And even among themselves*,* they are looking for some*” (Farmer 17, Antandroy).

Although migrants may theoretically mobilize broader networks [36] this advantage appears limited in this context: the quantity of cuttings required entails significant logistical costs (transport, etc.), and farmers tend to favor procurement within a narrow spatial radius. These dynamics point to differences in social capital that may influence access to resources circulating through relational networks, such as cassava cuttings, whose availability on markets is low and whose acquisition represents a substantial financial investment.

### Cultural differences and cultivated diversity

Beyond differences attributable to socio-economic characteristics, our results suggest that differentiated social representations of labor, hardship, the temporality of needs, and risk also shape crop diversity within each ethnolinguistic group.

As illustrated by the case of tobacco cultivation, the ethnolinguistic groups studied exhibit distinct agro-economic strategies structured around contrasting temporal horizons. Tandroy farmers frequently mentioned motivations linked to the agricultural calendar: they seek species and varieties with short cycles and staggered harvests, and avoid long-cycle crops, with the aim of securing regular income and food supply. This orientation appears closely tied to their cultural anchoring: Tandroy farmers, often perceived as migrants maintaining strong ties with their region of origin, operate within a logic of regularly sending resources to their families [54]. As some farmers explained:

“*When Tandroy are here*,* it is not without reason […] they are here to look for the* ravinaity (seeking a livelihood on non-ancestral land) *[…] they choose crops that will not take long to harvest […] that give them money quickly to send back home*” (Farmer 24, Masikoro);

“*Everything we do here is to bring it back there*” (Farmer 17, Antandroy).

In contrast, Masikoro farmers appreciate selling their harvest all at once, allowing them to obtain a large sum of money. This strategy may be a legacy of the cotton boom, when sales for the first time provided farmers with substantial liquidity immediately after harvest [46], which was then used to purchase livestock. The accumulation of cattle is a marker of social success among Masikoro people, with the sacrifice of many heads during ceremonies associated with health and prosperity [56].

Moreover, Masikoro farmers more frequently emphasize the arduousness and time requirements associated with certain crops (e.g., mung bean, cotton) and with maintaining a diversified range of species. Among Tandroy farmers, such discourse primarily concerns tobacco, which is widely recognized as particularly demanding to cultivate and is grown only rarely by Masikoro farmers partly for this reason. The literature portrays Tandroy people as hard-worker and deeply committed to agricultural work [43], which may help explain why they refer less often to physical hardship in their narratives. Their status as migrants in the area, and notably the regular remittance of resources to families remaining in their region of origin, may also encourage a stronger investment in labor-intensive crops.

Finally, the two ethnolinguistic groups appear to adopt differentiated risk-management strategies, as documented in other contexts [31]. Masikoro farmers rarely mention relying on cultivated diversity to cope with shocks; instead, they tend to favor species valued for their price stability (e.g., sweet potato, lima bean). Tandroy farmers, in contrast, more actively mobilize species diversification as a strategy to mitigate hazards. This may reflect agricultural practices developed in Androy, a region characterized by pronounced climatic variability, as highlighted by one farmer: “*In Androy*,* when we say there is no water*,* it means that for an entire year*,* there has not been any rain*,* maybe a single day of rain during the whole year*” (Farmer 17, Antandroy).

In such environments, farmers have historically relied on agroecological practices, including agroforestry, crop rotations, and crop-livestock integration, often cultivating multiple species within the same field, either in distinct plots or intercropped, to reduce risks associated with drought [72]. It is plausible that Tandroy farmers, having internalized these adaptive strategies, continue to apply them in the study area where we observed widespread intercropping practices. In other contexts, such as Thailand or Ghana, migration has been shown to facilitate the transfer of climate-adaptive innovations, particularly those compatible with small-scale, resource-constrained farming systems [36, 73]. In Australia, Klocker et al. (2018) argue, migrant farmers’ desire to grow culturally important crops in new environments, combined with their prior experience under diverse climatic conditions, constitutes a “*poorly recognised adaptive resource”* that expands the suite of available options [74]. This suggests that Tandroy farmers may act as transmitters of adapted agroecological knowledge.

### Limits and perspectives

Our study shows that, although both ethnolinguistic groups exhibit similar levels of species and varietal richness and share a core set of cultivated crops, specific differences consistently remain between them. These contrasts appear to arise from intertwined cultural and socio-economic factors. Other studies should seek to integrate additional socio-economic indicators such as family and hired labor, off-farm income activities, and other livelihood sources, to better understand how these dimensions interact. Likewise, accounting for the area allocated to each species and variety, despite the difficulty of collecting such data in intercropped systems [75], would allow a more fine-grained assessment.

By focusing on field crops and excluding wild and border plants, this study specifically targets agricultural knowledge rather than environmentally driven foraging practices, complementing work conducted in more diversified agroecosystems like homegardens or agroforestry systems [13, 38, 76]. Overall, our results clearly indicate that ethnolinguistic groups influence crop choices, preferences, and motivations to diversify (or not), underscoring the need for further research on how cultural and socio-economic drivers shape cultivated diversity.

Finaly, this study also highlights the absence of marked cultural segmentation in the management of cultivated diversity, in contrast to other contexts where social barriers can limit interactions between groups [17, 38]. Instead, and consistent with the observations of Brush and Perales [40], the two ethnolinguistic groups present in the study area do not appear to operate as closed systems: ideas, seeds, farming practices, and people circulate, while each group nevertheless maintains certain specific features. It is likely that, even if social boundaries may have existed in the past, the long-term settlement of Tandroy migrants, sometimes for more than seventy years [45], has progressively softened these divisions. Further research is needed to better understand the nature of social interactions between farmers from both ethnolinguistic groups (e.g., intermarriage) and their implications for agricultural dynamics, particularly regarding how they shape the circulation of plants and knowledge, and how this participates to enhance the resilience of farms and communities.

## Conclusion

To our knowledge, this is the first study to jointly examine how socio-economic and cultural factors linked to farmers’ ethnolinguistic belonging influence crop diversity. It complements the few existing works comparing crop choices across cultural groups and shows that farmers may not share the same motivations for diversifying species and varieties. For instance, we show that how crop diversity is perceived as a risk mitigation motivation differ between ethnolinguistics groups. These findings highlight the importance of considering both the characteristics and viewpoints of different cultural groups when designing initiatives for cultivated-diversity conservation and sustainable agricultural development supporting crop diversification [31, 35].

## Data Availability

The data used in this study are not publicly accessible to protect the privacy of individuals. However, they can be obtained from the corresponding author on reasonable request.
